# Spine Curvature Analysis between Participants with Obesity and Normal Weight Participants: A Biplanar Electromagnetic Device Measurement

**DOI:** 10.1155/2014/935151

**Published:** 2014-09-09

**Authors:** Manuel González-Sánchez, Jin Luo, Raymond Lee, Antonio I. Cuesta-Vargas

**Affiliations:** ^1^Department of Physiotherapy, Faculty of Health Sciences, University of Malaga, 29071 Málaga, Spain; ^2^Department of Life Sciences, Roehampton University, London SW15 4JD, UK; ^3^School of Clinical Sciences, Faculty of Health, Queensland University of Technology, Brisbane, QLD 4059, Australia

## Abstract

To analyse and compare standing thoracolumbar curves in normal weight participants and participants with obesity, using an electromagnetic device, and to analyse the measurement reliability. *Material and Methods*. Cross-sectional study was carried out. 36 individuals were divided into two groups (normal-weight and participants with obesity) according to their waist circumference. The reference points (T_1_–T_8_–L_1_–L_5_ and both posterior superior iliac spines) were used to perform a description of thoracolumbar curvature in the sagittal and coronal planes. A transformation from the global coordinate system was performed and thoracolumbar curves were adjusted by fifth-order polynomial equations. The tangents of the first and fifth lumbar vertebrae and the first thoracic vertebra were determined from their derivatives. The reliability of the measurement was assessed according to the internal consistency of the measure and the thoracolumbar curvature angles were compared between groups. *Results*. Cronbach's alpha values ranged between 0.824 (95% CI: 0.776–0.847) and 0.918 (95% CI: 0.903–0.949). In the coronal plane, no significant differences were found between groups; however, in sagittal plane, significant differences were observed for thoracic kyphosis. *Conclusion*. There were significant differences in thoracic kyphosis in the sagittal plane between two groups of young adults grouped according to their waist circumference.

## 1. Introduction

Obesity is one of the most serious and worrying health problems of our time and involves an increase in excess energy stored in the fatty tissues of the body [1], usually as a result of an imbalance between energy intake and expenditure [2, 3]. According to the World Health Organization (WHO) [2, 3], a person can be classified into six different categories according to his/her body mass index (BMI) calculated using the following formula: weight (kg)/height^2^ (m). These categories are participants with low weight, normal weight, overweight, and obesity types I-II-III. An individual is said to be obese if his/her BMI is greater than or equal to 30 kg/m^2^. However, there are some limitations when BMI is used to classify the population [4–6]. Another method has been proposed to identify obesity in individuals, which involves looking at the distribution of body fat [7] allowing the limitations of BMI to be overcome [7]. This index is based on the waist circumference (WC) and classifies an individual as a participant with obesity or a nonparticipant with obesity according to gender-specific thresholds, which are 102 cm and 89 cm for men and women, respectively [7]. These values are related to health risks in the same way as a BMI greater than or equal to 30 kg/m^2^ [7].

The excess fat that participants with obesity people must endure can essentially be considered dead weight attached to the body [8], which increases the mass of the different body segments and modifies physical geometry [9, 10].

The presence of this excess fat around the waist increases the muscular force required by the paravertebral musculature to maintain balance during the course of daily life activities in persons with obesity [11–14]. This modification of the internal forces of the trunk extended over time could justify the biomechanical changes (rebalancing of forces and changes in the curves) of the spine [10–14].

Maintenance of the thoracolumbar curvature is necessary to preserve the balance between both external forces that affect the participant and internal forces created by muscles [15], which could explain the increase in symptoms that are characteristic of low back pain suffered by people with obesity [16, 17]. Among the spines of normal weight participants, there is great variability in the thoracolumbar angles [18–20]. These differences reflect the variation in anthropometric and trunk morphological characteristics [21] and may also be influenced by the different measurement methodologies used across different studies. Measurements of spine curvature have been taken in cadavers [22]; however, these do not represent the curvatures found in standing living persons. In living participants, a device called a surface skin “spinal mouse” [23, 24] and X-rays [25–27] have been used. However, these have certain limitations that should be considered before use. The mouse spine is noninvasive and reliable but only gives the curvature of the spine in a single plane [24], while X-rays expose the patient to radiation and so should not be used as an instrument for clinical assessment.

An instrument that could overcome these limitations (exposure to radiation and measurement of spinal curvature in a single plane) is an electromagnetic device [28]. Keller et al. [29], Singh et al. [30], and Lundon et al. [28] have used this type of device for measuring thoracolumbar curves successfully in different types of people. For example, one study demonstrated significant differences in thoracolumbar curves between younger and older participants [28]. To the best of our knowledge, no study has used an electromagnetic device to measure differences in the thoracolumbar curvature between normal weight participants and participants with obesity. Therefore, the aim of this study was to analyse and compare the standing thoracolumbar curves of normal weight and obese participants using an electromagnetic device. Analysis of the measurement reliability of this instrument in these specific groups was also performed. The hypothesis of this study is that there are differences in the angles of the curves between these groups.

## 2. Materials and Methods

### 2.1. Design

This was a cross-sectional study, comparing thoracolumbar curvatures between participants with obesity and normal weight individuals.

### 2.2. Participants

A total of 36 individuals (18 men and 18 women) participated in the study. Study participants were recruited from staff and students at the University of Roehampton (London) and University of Malaga (Spain). Participants were divided into two groups (normal weight and participants with obesity) according to their WC. All participants whose WC was less than 102 cm (men) or 89 cm (women) were part of the normal weight group (nonobese participants), whereas all participants with values greater than or equal to the obesity thresholds were included in the participants with obesity group [7].

The inclusion criteria used were normal weight young adults (less than 40 years) of both genders. These inclusion criteria were defined in order to minimise the effects of age at the time of thoracolumbar curvature measurement. Exclusion criteria were the presence of musculoskeletal or neurological disorders, a history of spinal or hip surgery, pregnancy, cancer, and osteoporosis. Consequently, any participants with an orthopaedic implant (a medical device that replaces a whole or part of a joint) or an electrically powered medical implant (i.e., a pacemaker, implantable defibrillator, cochlear implant, and insertable cardiac neurostimulator or monitor) were excluded. Moreover, with the intention of better delineating both groups of young healthy participants compared in the present study, all volunteers who did not fall within the BMI ranges >18 kg/m^2^ and ≤25 kg/m^2^ and >30 kg/m^2^ and ≤35 kg/m^2^ were excluded from the study.

### 2.3. Ethical Considerations

Ethical approval for the study was granted by the Ethics Committee of Roehampton University (UK) and by the Faculty of Health Sciences at the University of Malaga (Spain). Data were handled in accordance with the Ethical Standards of the Helsinki Declaration of 1975, as revised in 2008 [31]. Each participant gave written informed consent before the experiment. Each participant was able to leave the study at any time at their own request. The protection of personal data was in accordance with the Spanish Organic Law on Personal Data Protection 19/55.

#### 2.3.1. Electromagnetic Tracking Device

Spinal curvature was measured using an electromagnetic tracking device (Fastrak, Polhemus). This device works with a sample frequency of 120 Hz and a static accuracy position of 0.76 mm for the* X*,* Y*, or* Z* position and a static accuracy orientation of 0.15° RMS [32].

#### 2.3.2. Protocol

The protocol applied in this study was first used and described in detail by Lundon et al. [28].

### 2.4. Participant's Position

Participants stood in an upright position and were relaxed, looking forward with their knees straight and arms parallel to the body and a distance of 25 cm between their feet; they maintained expiratory apnoea whilst performing the various steps of the protocol.

### 2.5. Protocol: Part One

The protocol was divided into two phases. In the first phase, the examiner marked various reference points (T_1_–T_8_–L_1_–L_5_ and both PSIS (posterior superior iliac spines)) by palpating the different points along the spine; these points were digitised. The same examiner measured the reference points three times consecutively.

The examiner then conducted a trace from T_1_ to L_5_, which was repeated three times, uniting all spinous processes lying between these points. For this purpose, a small probe of nonmetallic material was attached to the electromagnetic sensor, making the measurement more accurate and resulting in a more comfortable sensation on the skin of the participant.

### 2.6. Protocol: Part Two

In the second part of the protocol, the local system was established using reference marks of the PSIS and T_8_, the origin of which is located at the midpoint between the two PSIS. By performing a transformation from the global coordinate system created from the source to the local Fastrak system explained above, the thoracolumbar curves could be adjusted by fifth-order polynomial equations. The tangents of the first and fifth lumbar vertebrae and the first thoracic vertebra were determined from their derivatives.

For both the coronal and sagittal planes, dorsal kyphosis was calculated by measuring the angle created at the intersection of the two tangents T_1_ and passing through L_1_. Additionally, the lumbar lordosis was calculated from the angle created at the intersection of the tangents that passed along L_1_ and L_5_.

To perform the calculations explained, digitised reference points (spine and PSIS), the data recorded during the sliding of the electromagnetic sensor from T_1_ to L_5_ and an algorithm created in MATLAB software (MATLAB 7.11 R2010b) were used. Lumbar lordosis (sagittal plane) and left convexity (coronal plane) were considered positive values.

### 2.7. Reliability Procedure

To measure the reliability of the procedure, each part of the study was conducted three times: scanning each of the benchmarks used to calculate the curvature ((T_1_–T_8_–L_1_–L_5_ and both PSIS (posterior superior iliac spines)), slipping of the electromagnetic device from T_1_ to L_5_, and finally applying the algorithm of MATLAB software used for the calculation of the curvatures in the two planes.

Reliability calculation was made on the curvature measures of both planes studied, as they comprise the entire measurement protocol (use of electromyographic device for recording data in the back and an algorithm using MATLAB for calculating curvatures).

Reliability was considered as a test-retest standard deviation of differences or as the 95% limits of agreement [33, 34] and was performed only by a researcher; therefore, the reliability calculated was intraobserver.

### 2.8. Data Analysis

A descriptive analysis of the sample was performed. Subsequently, using the results obtained from the digitisation of points as a description of the spine, the reliability of the measurement was assessed according to the internal consistency of the measure (Cronbach's alpha) with a 95% confidence interval (CI). Finally, a comparison of the thoracolumbar curvature angles was performed between groups, using Student's* t*-test as a parametric test for independent data and Wilcoxon's test for nonparametric tests. Analysis of normality of the sample was carried out using a Kolmogorov-Smirnov test before comparing variables between groups. The level of significance was set at *P* < 0.05.

The statistical package for social sciences (SPSS) (version 17.0 for Windows, Illinois, USA) was used to perform the data analysis.

## 3. Results

A total of 36 participants were divided into two groups: G_normal  weight_: 18 (9 men and 9 women) and G_participants  with  obesity_: 18 (9 men and 9 women).  Table 1 shows the descriptive data of the sample. No significant differences between the two groups were observed for any of the measured variables except for WC, BMI, and weight.

The reliability of the measurement of the curvature of the back was high. Cronbach's alpha ranged from 0.896 to 0.918 in the sagittal plane and from 0.824 to 0.862 in the coronal plane. The other measures of reliability are shown in Table 2.

Table 3 shows the differences in the means of the different curvatures, in both the sagittal and the coronal plane. No significant differences were found between groups in the coronal plane; however, significant differences were observed for thoracic kyphosis and the curvature described by the spine between T_1_ and T_8_.

On the other hand, Table 4 shows the mean values and the differences between groups. It can be observed that, in the group of nonparticipants with obesity, there is a significant difference in the lumbar lordosis, while in the subset of participants with obesity, a significant difference was found for thoracic kyphosis.

**Figure 1:**
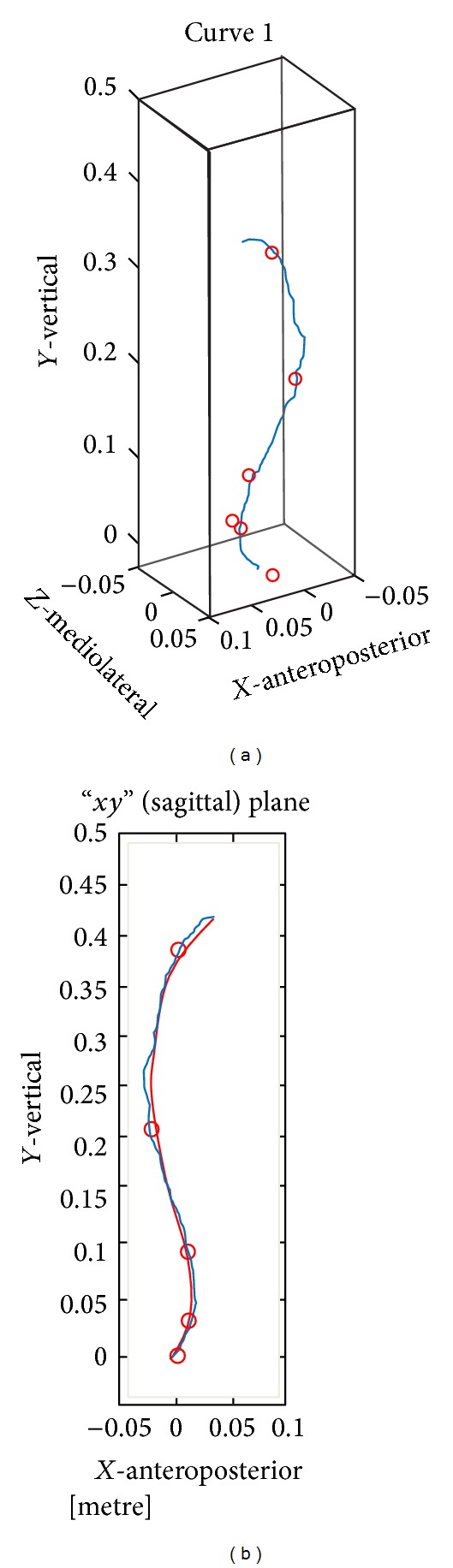
Perspective of both curves integrated in three dimensions.

Figure 1 shows an example of the reference points used to measure the coronal and sagittal planes of the bending angles, which determine the thoracolumbar curvature, and the results of the perspective of both curves integrated in three dimensions.

## 4. Discussion

This study is the first to measure the differences in thoracolumbar curves (in both sagittal and coronal planes) between participants with obesity and normal weight participants using an electromagnetic device that describes the curvature in two dimensions. After analysing the results, the hypothesis was partially confirmed, as significant differences were observed in the thoracolumbar curvatures, but only in thoracic segments. No significant differences were observed in the lumbar region for either plane analysed.

The results obtained in this study are consistent with another study that used the same methodology for measuring curvature but which compared young participants with older participants [28]. Both studies included groups with a comparable BMI, with a mean result (±standard deviation) of 21.47 kg/m^2^ (±2.36) in the present study and 22.48 kg/m^2^ (±2.51) in study by Lundon et al. [28]. The mean angles of the curvatures in the sagittal plane measured by Lundon et al. [28] were −38.82° (±9.86) for thoracic kyphosis and 30.37° (±8.33) for lumbar lordosis, respectively; these were similar to the values obtained in the present study, which were −38.59° (±2.71) and 28.02° (±3.91), respectively. Similarly, the mean angles of the curvatures in the coronal plane in the study by Lundon et al. [28] were 0.12° (±7.27) for thoracic kyphosis and 0.83° (±3.49) for lumbar lordosis; these were similar to the values found in the present study, which were 0.27° (±0.60) and 0.12° (±0.32), respectively (Table 3).

The difference in these values may be attributed to interobserver variability [18–20, 28]. In addition, both studies observed significant differences in the thoracic curvature between groups. In the study of Lundon et al. [28], young normal weight participants were compared with older participants, while in the present study, young normal weight participants were compared with young participants with obesity. However, in both studies, no significant differences were found in any of the lateral curvature measures in the coronal plane.

The main finding of this study was that there were significant differences in thoracic kyphosis between normal weight individuals and participants with obesity. This difference was statistically significant between T_1_ and T_8_. This may be due to increased spinal curvature as a result of an increase in upper limb mass [9, 10], as well as the increased muscle strength required to rebalance the body [8, 12, 13], which indicates that increased thoracic kyphosis would be one of the first consequences of rebalancing forces that are exhibited by participants with obesity. This biomechanical rebalancing suffered by participants with obesity people could cause a balance deficit and reduced performance in functional tasks of daily living [8]. It may be possible to find an explanation in older people that can partly explain why there was some similarity between the curves measured in the present study and those measured in the study of both young and old participants with obesity [28], such that significant differences were observed in the curvature of the thoracic spine.

Furthermore, this study found no significant differences in the thoracolumbar curvature in the coronal plane between participants with obesity and normal weight participants.

In this study, it was acknowledged* a priori* that the curvature measurements were limited by the presence of subcutaneous adipose tissue in participants with obesity. Therefore, analysis of the reliability of the measure was warranted. Reliability in the sagittal plane was 0.896 (T_8_–L_1_), while in the coronal plane, reliability ranged between 0.824 and 0.862. This degree of reliability was in line with the reliability presented by Lundon et al. in young and old normal weight individuals [28].

On the other hand, if the data are analysed following stratification by gender, the angle of the column changes in both men and women (Tables 3 and 4). Specifically, in women, as seen in the participants with obesity group, the natural curve of the lumbar lordosis is maintained (difference of 0.37°); however, the thoracic kyphosis increases its curve by 18.85°, being especially pronounced in the first segment T_1_–T_8_ (+21.67°), which implies that the thoracic spine gets flatter in women with obesity. Furthermore, men with obesity suffered a structural change in the two curves, increasing the angle of thoracic kyphosis (+12.15°) and reducing the lumbar lordosis angle (+6.43°) (Table 3). It is observed that obesity tends to match the curves of the spine between men and women in both thoracic kyphosis and lumbar lordosis (Table 4).

This study had some weaknesses, including the absence of a reference used in routine practice such as radiography in two planes, which was excluded herein to avoid radiation to the participants, but would provide improved criterion validity. Furthermore, the study sample was small. However, one of the main strengths of the study was the novelty of using this methodology in participants with obesity, encouraging its use in consolidating clinical practice. Future studies should be conducted in other groups as well as in a greater number of individuals, expanding the sample presented in this study.

## 5. Conclusion

The main conclusion of this study is that when using an electromagnetic device to measure thoracolumbar curvature, there were significant differences in thoracic kyphosis in the sagittal plane between the two groups of young adults grouped according to their waist circumference (participants with obesity and normal weight participants). This method allows analysis of curvature in the sagittal and coronal planes with a single measurement. No significant differences were observed in the other curvatures analysed (lumbar lordosis in the sagittal plane and thoracic and lumbar curvature in the coronal plane). Furthermore, the reliability of the use of an electromagnetic device for measuring the two-dimensional curvature in obese individuals and individuals with normal weight has been excellent.

## Figures and Tables

**Table 1:** Descriptive characteristics of the two groups in the study.

	Normal weight	Participants with obesity	Mean difference (95% CI)	Sig. (bilat.)
Age (y)	**30.94** (±4.45)	**30.29** (±4.46)	**0.65** (−2.41/3.72)	**0.669**

Weight (kg)	**59.42** (±9.97)	**94.05** (±11.55)	**−34.63** (−42.08/−27.18)	**0.000**

Height (cm)	**165.93** (±7.82)	**170.28** (±9.26)	**−4.34** (−10.26/1.58)	**0.145**

BMI (kg/m^2^)	**21.47** (±2.36)	**32.34** (±1.70)	**−10.87** (−12.28/−9.46)	**0.000**

WC (cm)	**77.53** (±7.08)	**101.08** (±9.63)	**−23.54** (−29.41/−17.67)	**0.000**

WC: waist circumference.

**Table 2:** Measures of reliability of the different spinal segment's angles.

	Measure	Cronbach's alpha	95% Confidence interval	
			Inferior	Superior
Sagittal plane	T_1_–L_1_	0.903	0.878	0.929
	T_1_–T_8_	0.906	0.883	0.928
	T_8_–L_1_	0.896	0.863	0.918
	L_1_–L_5_	0.918	0.903	0.949

Coronal plane	T_1_–L_1_	0.824	0.796	0.847
	L_1_–L_5_	0.862	0.834	0.901

**Table 3:** Comparison of thoracolumbar curvatures in the coronal and sagittal plane.

Curvature	Men			Women			Group		
	Normal weight	Participants with obesity	Diff.	Normal weight	Participants with obesity	Diff.	Normal weight	Participants with obesity	Diff.
Sag. thoracic kyphosis (deg)	**39.65** (±2.09)	**51.80** (±1.88)	**12.15** ∗∗∗ (±1.03)	**38.05** (±2.91)	**56.18** (±3.43)	**18.85** ∗∗∗ (±1.42)	**38.59** (±2.71)	**54.20** (±3.43)	**15.61** ∗∗∗ (±1.10)

Sag. angles T_1_–T_8_ (deg)	**26.40** (±1.79)	**43.07** (±1.56)	**16.67** ∗∗∗ (±0.89)	**25.64** (±2.09)	**44.32** (±2.86)	**21.67** ∗∗∗ (±1.10)	**25.90** (±1.97)	**45.07** (±3.09)	**19.17** ∗∗∗ (±0.87)

Sag. angles T_8_–L_1_ (deg)	**16.03** (±1.12)	**8.94** (±0.74)	**7.09** ∗∗∗ (±0.47)	**13.31** (±3.50)	**9.34** (±0.42)	**3.96** ∗∗∗ (±0.38)	**14.21** (±1.67)	**9.13** (±0.62)	**5.08** ∗∗∗ (±0.43)

Sag. lumbar lordosis (deg)	**31.84** (±1.52)	**25.41** (±1.11)	**6.43** ∗∗∗ (±0.68)	**26.11** (±3.26)	**25.74** (±0.43)	**0.37** (±0.95)	**28.02** (±3.91)	**26.57** (±0.86)	**1.46** (±0.97)

TCCP (deg)	**−0.00** (±0.65)	**0.07** (±0.71)	**0.07** (±1.03)	**0.10** (±0.60)	**−0.20** (±0.58)	**0.31** (±0.27)	**0.27** (±0.60)	**−0.06** (±0.64)	**0.33** (±0.41)

LCCP (deg)	**−0.22** (±0.18)	**0.21** (±0.32)	**0.43** (±0.15)	**0.09** (±0.32)	**−0.02** (±0.37)	**0.10** (±0.16)	**0.12** (±0.32)	**−0.16** (±0.35)	**0.28** (±0.36)

Sag: sagittal plane.

TCCP: thoracic curvature in coronal plane.

LCCP: lumbar curvature in coronal plane.

Signification level:

*P ≤ 0.05.

**P ≤ 0.01.

***P ≤ 0.001.

**Table 4:** Comparison of thoracolumbar curvatures in the coronal and sagittal planes separate by group.

Curvature	Nonparticipants with obesity			Participants with obesity		
	Men	Women	Diff.	Men	Women	Diff.
Sag. thoracic kyphosis (deg)	**39.65** (±2.09)	**38.05** (±2.91)	**1.60** (±1.34)	**51.80** (±1.88)	**56.18** (±3.43)	**5.10** ∗∗ (±1.32)

Sag. angles T_1_–T_8_ (deg)	**26.40** (±1.79)	**25.64** (±2.09)	**0.76** (±0.99)	**43.07** (±1.56)	**44.32** (±2.86)	**4.24** ∗∗ (±1.10)

Sag. angles T_8_–L_1_ (deg)	**16.03** (±1.12)	**13.31** (±3.50)	**2.73** ∗∗∗ (±0.52)	**8.94** (±0.74)	**9.34** (±0.42)	**0.40** (±0.30)

Sag. lumbar lordosis (deg)	**31.84** (±1.52)	**26.11** (±3.26)	**5.73** ∗∗∗ (±1.42)	**25.41** (±1.11)	**25.74** (±0.43)	**−0.33** (±0.40)

TCCP (deg)	**0.00** (±0.65)	**0.10** (±0.60)	**−0.11** (±0.31)	**0.07** (±0.71)	**−0.20** (±0.58)	**0.27** (±0.32)

LCCP (deg)	**−0.22** (±0.18)	**0.09** (±0.32)	**−0.31** (±0.14)	**0.21** (±0.32)	**−0.02** (±0.37)	**0.23** (±0.17)

Sag: sagittal plane.

TCCP: thoracic curvature in coronal plane.

LCCP: lumbar curvature in coronal plane.

Signification level:

*P ≤ 0.05.

**P ≤ 0.01.

***P ≤ 0.001.
